# Altered mastication adversely impacts morpho-functional features of the hippocampus: A systematic review on animal studies in three different experimental conditions involving the masticatory function

**DOI:** 10.1371/journal.pone.0237872

**Published:** 2020-08-20

**Authors:** Maria Grazia Piancino, Alessandro Tortarolo, Antonella Polimeni, Ennio Bramanti, Placido Bramanti

**Affiliations:** 1 Department of Surgical Sciences, Dental School, University of Turin, Turin, Italy; 2 Department of Oral and Maxillo-Facial Science, Sapienza University of Rome, Rome, Italy; 3 Department of Biomedical and Dental Sciences, Morphological and Functional Images, University of Messina, Messina, Italy; 4 IRCCS Centro Neurolesi "Bonino Pulejo", Messina, Italy; University of Modena and Reggio Emilia, ITALY

## Abstract

Recent results have established that masticatory function plays a role not only in the balance of the stomatognathic system and in the central motor control, but also in the trophism of the hippocampus and in the cognitive activity. These implications have been shown in clinical studies and in animal researches as well, by means of histological, biochemical and behavioural techniques. This systematic review describes the effects of three forms of experimentally altered mastication, namely soft-diet feeding, molar extraction and bite-raising, on the trophism and function of the hippocampus in animal models. Through a systematic search of PubMed, Embase, Web of Science, Scopus, OpenGray and GrayMatters, 645 articles were identified, 33 full text articles were assessed for eligibility and 28 articles were included in the review process. The comprehensiveness of reporting was evaluated with the ARRIVE guidelines and the risk of bias with the SYRCLE RoB tool. The literature reviewed agrees that a disturbed mastication is significantly associated with a reduced number of hippocampal pyramidal neurons in Cornu Ammonis (CA)1 and CA3, downregulation of Brain Derived Neurotrophic Factor (BDNF), reduced synaptic activity, reduced neurogenesis in the Dentate Gyrus (DG), glial proliferation, and reduced performances in behavioural tests, indicating memory impairment and reduced spatial orientation. Moreover, while the bite-raised condition, characterized by occlusal instability, is known to be a source of stress, soft-diet feeding and molar extractions were not consistently associated with a stress response. More research is needed to clarify this topic. The emerging role of chewing in the preservation of hippocampal trophism, neurogenesis and synaptic activity is worthy of interest and may contribute to the study of neurodegenerative diseases in new and potentially relevant ways.

## Introduction

Recent research results have established that the masticatory function plays a role not only in the balance of the stomatognathic system and in the central motor control, but especially in the development of the cognitive activity and in the slowdown of the unavoidable cognitive decay. These unsuspected results have been clearly shown by basic research with histomorphological outcomes and behavioural tests, as well as in clinical studies [1, 2].

The importance of the masticatory function on maxillary growth, on the balance of the stomatognathic system and on the central motor control is well known in literature [3–6].

Chewing, one of the phylogenetically oldest functions of the stomatognathic system, is a complex, highly coordinated and continuously modulated movement, able to constantly adapt to the volume and consistency of the alimentary bolus through a diversity of masticatory patterns, emerging out of the central integration of a great number and variety of peripheral inputs [7, 8]. The human masticatory function is a symmetrical, rhythmic and semi-automatic movement that alternates the involvement of the two sides of the dental arches. The teeth, no longer necessary for our survival as they are for the rest of the animal kingdom, play nevertheless a crucial role in the coordination and harmonious execution of the masticatory function, which, in turn, influences the development of the craniofacial region. While it is well known that the motor control of the masticatory function involves a large portion of the central nervous system, including brainstem, cerebellum, basal nuclei, midbrain and cortex, its influence on the hippocampus, memory and cognitive activity has only recently emerged [9].

In the last few years, the relationship between tooth loss and cognitive decline in the elderly has been established in a number of clinical studies which have been critically reviewed [1]. This relationship has been significantly shown in laboratory animals as well, highlighting the morphological alterations of the hippocampus and the related behavioral outcomes. Animal studies enable us to observe simultaneously the outcomes of behavioral tests and the histological and biochemical alterations associated with experimentally induced masticatory disturbances. Young, middle-aged and senile mice subjected to different forms of masticatory imbalances show a lower number of hippocampal neurons, an increased number of astrocytes and reduced progenitor cell proliferation in the hippocampal dentate gyrus (DG) [2].

In order to alter the masticatory function, different animal models have been exposed to three experimental conditions: soft-diet feeding, molar extraction and occlusal disharmony (bite-raising). These approaches shed light on the fact that masticatory dysfunction, in addition to the already cited histological effects on the number of neurons, alters the biochemical and hormonal balance and impairs spatial learning and memory [2].

Descriptive revues, mainly clinical reviews, have recently been published due to the interest of the topic [6, 10–12]. It is worth describing in a rigorous way the picture that is emerging from animal studies in order to clarify how the different experimental conditions allow us to understand the association between mastication and cognition.

This systematic review aims at evaluating the literature on the influence of three forms of experimental alteration of the masticatory function, namely soft-diet feeding, molar extraction and bite-raising, on the histochemistry, function and behavioral role of the hippocampus in laboratory animals.

## Materials and methods

### Pre-clinical PICO and rationale for the review

In animal research, it is possible to experimentally alter the masticatory function in a controlled way and to assess its effects objectively. For this systematic review, the pre-clinical PICO was: “What is the impact of an experimentally altered masticatory function (I), compared to an undisturbed masticatory function, (C) in laboratory animals (rats and mice) (P) on the histology and biochemistry of the hippocampus (O)?”.

There was no significant deviation from the initial purpose, which was generally maintained until the end of the review. However, after the first evaluation of the published literature, it appeared clear that it was important to separately describe the different experimental conditions. This planning allowed for a more accurate evaluation of the results.

### Search strategy

A search of PubMed, Embase, Web of Science, Scopus, OpenGray and GrayMatters was conducted until July 2020, limiting the search to studies published in English within the last 10 years, as reported in Table 1. Additional studies were taken from reference lists of previous review articles and citations of relevant original articles were screened. References of included studies were checked by a research librarian.

**Table 1:** Search strings and results.

Database	Search string	Number of results
*PubMed*	(("Mastication"[Mesh] OR masticat* OR chew*) OR (bite-rais*) OR (("Tooth Extraction"[Mesh] OR tooth extract* OR molar extract*) OR molarless) OR (liquid diet OR soft diet OR hard diet OR powdered diet OR hard bolus OR soft bolus) AND ("Hippocampus"[Mesh] OR hippocampus))	167
*Embase*	('hippocampus'/exp OR hippocampus) AND (('mastication'/exp OR mastic* OR chew*) OR ('tooth extraction'/exp OR 'tooth extract*' OR 'teeth extract*' OR 'molar extract*' OR molarless) OR (('bite rais*' OR bite) AND rais*) OR ('soft diet'/exp OR 'liquid diet'/exp OR 'soft diet' OR 'hard diet' OR 'liquid diet' OR powdered diet))	206
*Web of Science*	((hippocampus) AND ((masticat* OR chew*) OR (bite-rais* OR bite rais* OR tooth extract* OR teeth extract* OR molar extract* OR molarless OR soft diet OR hard diet OR liquid diet OR powdered diet)))	174
*Scopus*	((hippocampus) AND ((masticat* OR chew*) OR (bite-rais* OR bite rais* OR tooth extract* OR teeth extract* OR molar extract* OR molarless OR soft diet OR hard diet OR liquid diet OR powdered diet)))	98
*OpenGray*	((hippocampus) AND ((masticat* OR chew*) OR (bite-rais* OR bite rais* OR tooth extract* OR teeth extract* OR molar extract* OR molarless OR soft diet OR hard diet OR liquid diet OR powdered diet)))	0
*GrayMatters*	((hippocampus) AND ((masticat* OR chew*) OR (bite-rais* OR bite rais* OR tooth extract* OR teeth extract* OR molar extract* OR molarless OR soft diet OR hard diet OR liquid diet OR powdered diet)))	0

#### Search results

**Fig 1:**
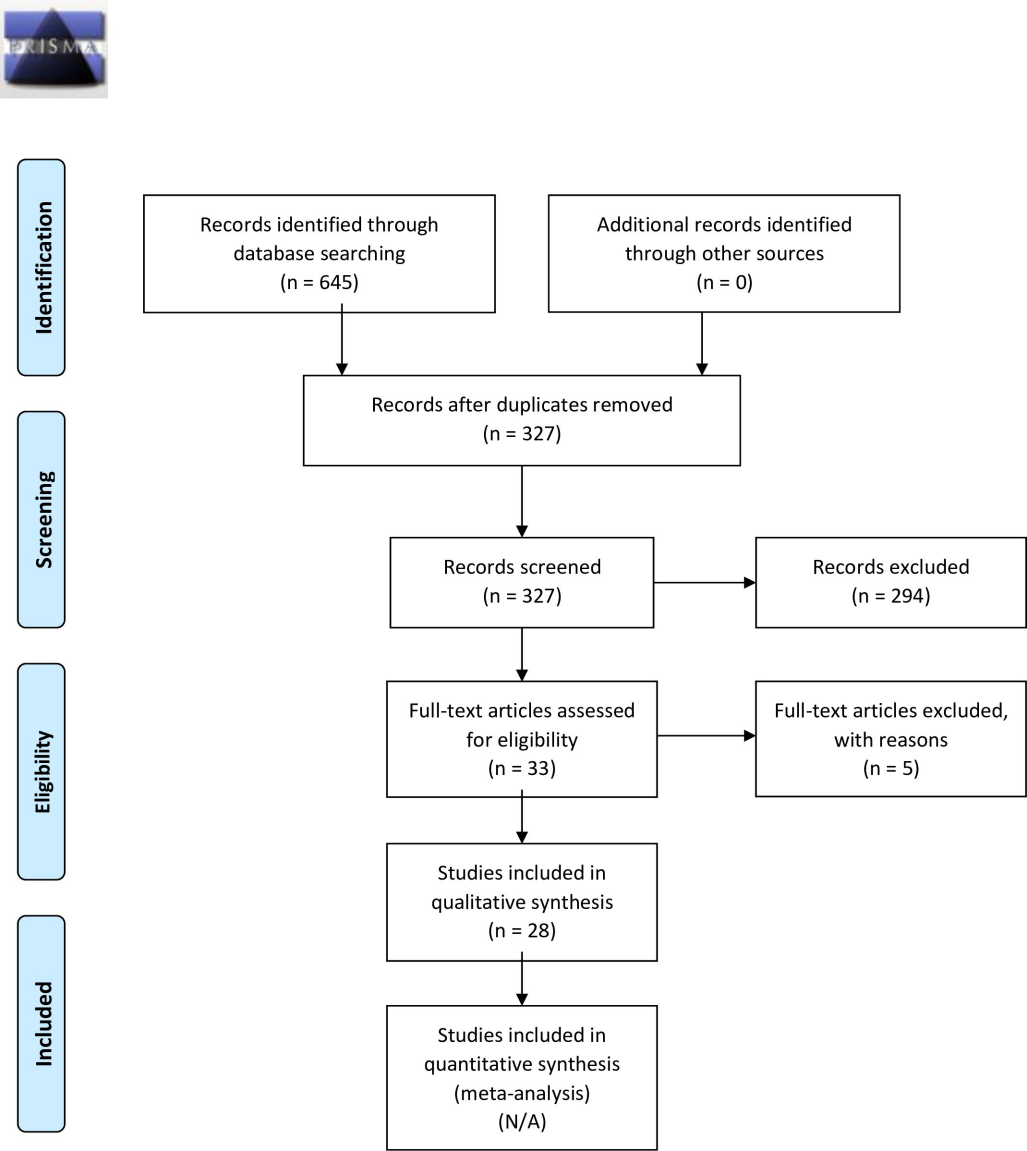
PRISMA 2009 flow diagram.

645 articles were identified through database searching. After removing the duplicates, 327 articles were screened by reviewing the abstracts. 33 full text articles were assessed for eligibility and, after 5 exclusions, 28 articles were included in the review process as reported in Fig 1.

#### Selection procedure

After removal of the duplicates, articles were screened independently by AT and EB; when opinions about inclusion differed, a third person (MGP and/or PB and/or AP) was consulted.

### Inclusion criteria

Inclusion criteria were the following:

- Type of study: animal intervention study.
- Subjects: laboratory animals.
- Control group: present.
- Experimental stimulus: alteration of the masticatory function.
- Outcomes: histological or biochemical analysis of the hippocampus.

Exclusion criteria were the following:

- Molars cut off at gingival level: to reduce the possible confounding effects related to pulpal inflammation and retention of periodontal receptors, molarless animal models obtained by means of cutting off the tooth crown at the level of the gingiva were not included.
- Cast splints or crown: models of bite-raising obtained by cast splints or crowns were not included because of the possible confounding effects related to pulpal inflammation after poor tooth preparation.
- Unilateral molar extractions: unilateral models of both molar extraction and bite-raising were not included in order to avoid the disturbing biomechanical effects and related functional alterations, due to an asymmetric dental status, which is hardly comparable to a symmetric one.
- Hard and soft-diet feeding: studies were included only if the differences in the texture and hardness of the different types of food were clearly outlined. In particular, studies that compared standard pellet chow with harder diets were excluded unless the physical characteristics of the harder foodstuff were rigorously described.

Studies presenting behavioral outcomes alone were not included, but behavioral outcomes were discussed if presented in association with histological or biochemical ones.

### Classification of the selected studies

The studies selected by the review process have been divided in three groups according to the experimental conditions: 1) studies on soft-diet feeding; 2) studies on tooth extraction; 3) studies on bite-raising.

### Comprehensiveness of scientific reporting and risk of bias

The comprehensiveness of scientific reporting in included studies was assessed with the ARRIVE checklist [13] (see S1 Table) by AT and EB. The risk of bias of included studies was assessed with the SYRCLE tool for risk of bias in animal studies [14] (see Tables 2 and S2) by AT and AP. For each study, an evaluation was made for each item in the SYRCLE tool. A general assessment was carried out by weighting the results of the different questions in each bias domain, in order to provide the best summary of the evidence that the authors could provide. This assessment reflects a criterion of thoughtful evaluation rather than an arithmetical one. S2 Table reports the answers of individual included studies for each question in the SYRCLE tool. In both instances, the work was supervised by MGP and PB, who were consulted when opinions differed.

**Table 2:** Risk of bias assessment.

Bias domain	Assessed risk of bias
*Selection bias*	Unclear
*Performance bias*	Unclear
*Detection bias*	Unclear
*Attrition bias*	Low
*Reporting bias*	Low
*Other bias*	Low

### Data extraction

The analytical evaluation of included studies was carried out. Subsequently, the data obtained was combined to produce three analytical tables and one summary table.

## Results

The following studies met the inclusion criteria: 1) studies on soft-diet feeding [2, 15–22]; 2) studies on tooth extraction [23–36]; 3) studies on bite-raising [37–41].

The comprehensiveness of scientific reporting of included studies assessed with the ARRIVE checklist [13] is described in S1 Table. While the level of agreement with the checklist was generally high, most articles lacked information concerning items 11, 14 and 17. In other words, most studies failed to report the details concerning allocation of animals to experimental groups, baseline data and adverse events (or to state explicitly that no adverse event took place; this may however be inferred from the stability of group numerosity from the beginning to the end of experimentation, as indeed was generally the case).

The risk of bias of included studies assessed with the SYRCLE tool for risk of bias in animal studies [14] is reported in Tables 2 and S2. While the risk of attrition bias, reporting bias or other bias could be adequately assessed, the risk of selection bias, performance bias and detection bias of included studies could not be assessed because, with very few exceptions, the necessary information (concerning randomisation of sequence generation, allocation concealment, randomisation of housing, blinding of caregivers, randomisation of outcome assessment and blinding of researchers assessing outcome) was not reported.

In the SYRCLE RoB tool, selection bias is investigated by means of three items (sequence generation, baseline characteristics and allocation concealment). While the majority of included studies reported sufficient information on baseline characteristics, none reported details of allocation concealment and a minority reported details of sequence generation: two out of three items, among them arguably the most important of the three (sequence generation, i.e. the presence of a random element in the selection of experimental groups), were impossible to adequately assess. The risk of selection bias was therefore assessed as unclear. Detection bias is investigated by means of two items (random outcome assessment and blinding). The majority of included studies did not report information concerning the blinding of examiners, and none reported information about random outcome assessment. The risk of detection bias was therefore assessed as unclear. The risk of attrition bias, reporting bias and other bias was assessed as low, because a majority of included studies adequately reported this information.

The analytic results are presented in table forms, as follows:

- Table 3: Soft-diet feeding
- Table 4: Molar extraction
- Table 5: Bite-raising
- Table 6: Summary of results

**Table 3:** Soft-diet feeding.

Animal model and [N]	Age at stimulus application and [type of stimulus]	Age at sacrifice	Marker analyzed	Structure analyzed	Type of analysis	Corticosterone evalutation	Results
Akazawa, Y., et al. (2013). *International Journal of Molecular Medicine*, *31*(2), 307–314.							
C57BL/6 mice [not declared]	6 wk [normal pellet chow (group 1); pellet hardened by autoclave (group 2)].	20 wk.	Number of BrdU-, c-Fos- and nNOS-labeled cells in DG; volume of the DG; mRNA expression of growth factors, neurotrophic factors, NSC markers and receptors in the hippocampus; body weight; escape latency time in water maze and performance in probe test.	Hippocampus.	Immunohistochemistry; RT-PCR; body weight measurement; Morris water maze test and probe test in water maze.	Not investigated.	BrdU-, c-Fos and nNOS-positive cells in group 2 were significantly increased, as well as hippocampal volume. In the Morris water maze, mice in group 2 required significantly less time to reach the platform and spent significantly more time in the former platform area. The expression of glutamate receptor 1 mRNA, but not the other mRNAs investigated, was significantly increased in group 2. No significant difference in body weight was found.
Frota de Almeida, M., et al. (2012). *BMC Neuroscience*, *13*(1), 23.							
Albino Swiss mice [66]	3 wk [normal pellet chow (group 1); powdered pellet (group 2)].	3, 6 and 18 mo.	Escape latency, distance travelled, average swimming speed and trajectories in the Morris water maze; number of GFAP-labeled cells (astrocytes).	CA1.	Morris water maze test; immunohistochemistry; body weight measurement.	Not investigated.	Laminar distribution of astrocytes was variably, but significantly, affected. Worse performances in the water maze were recorded for group 2.
Fukushima-Nakayama, Y., et al. (2017). *Journal of Dental Research*, *96*(9), 1058–1066.							
Male C57BL/6J mice [63]	3 wk [normal pellet chow (group 1); powdered diet (group 2)].	14 wk.	Entry latency (PAT); sniffing time (OLT); time spent in central zone and distance traveled (OFT); average latency to fall (RT); GAPDH, BDNF and NTRK2 mRNA levels; BrdU-, c-Fos-, NeuN- and PSD95-labeled cells; body weight; masticatory muscles weight; volume and characteristics of maxillary and mandibular bones.	Hippocampus and cerebral cortex.	Passive avoidance test (PAT); object location test (OLT); open-field test (OFT); rotarod test (RT); RT-PCR; immunohistochemistry; body weight measurement; morphometry of the stomatognathic apparatus.	Not investigated.	Reduced mastication in group 2 was found to be associated with a decline in memory and learning function, with decreased neurogenesis in DG and reduced number of NeuN-labeled neurons in CA1 and CA3, neuronal activity and synapse formation, all of which are related to BDNF. The growth of the maxillofacial complex, but not that of the rest of the body, was found to be associated with reduced mastication.
Niijima-Yaoita, F., et al. (2013). *Neurochemistry International*, *63*(4), 309–315.							
Male Balb/c mice [not declared]	3 wk [pellet chow (group 1); powdered diet (group 2)].	20 wk.	Number of activity counts (SLA); total amount of time spent in interaction (SIT); dopamine (DA) turnover ratio; DA receptors mRNA levels; body weight.	Frontal cortex and hippocampus.	Spontaneous locomotor activity (SLA); social interaction test (SIT); high performance liquid chromatography and electrochemical detection; RT-PCR; effect of PD168077 on SIT; body weight measurement.	Not investigated.	Mice in group 2 showed hyper locomotor activity in the first 30 min of their exposure to the novel/unfamiliar environment; after habituation to a novel/unfamiliar environment, they exhibited an increased SI time; the powdered diet increased dopamine turnover and decreased D4 receptor mRNA expression in the frontal cortex, and D4 receptor agonist attenuated the increased SI time in group 2.
Nose-Ishibashi, K., et al. (2014). *Neuroscience*, *263*, 257–268.							
Male C57BL6/J mice [51]	3 wk [mice fed a hard diet for 4 weeks (group 1); mice fed a soft diet for 4 weeks (group 2); mice fed a hard diet for 11 weeks (group 3); mice fed a soft diet for 11 weeks (group 4); mice changed to a hard diet at 7 weeks of age after receiving a soft diet for 4 weeks (group 5)].	7 and 14 wk.	BDNF, TrkB, and Akt1 mRNA expression; number of BrdU-labeled cells; escape latency in water maze; body weight.	DG and frontal cortex.	RT-PCR; immunohistochemistry; Morris water maze; body weight measurement.	Not investigated.	At the age of 14 weeks the number of BrdU-labeled cells (hippocampal neural progenitor proliferation) was significantly lower in group 4 than in group 3. In mice initially given the soft diet and later changed to a hard diet (group 5), the number of BrdU-positive cells was decreased compared to group 3, but this decrease was not significant. No statistically significant differences were observed between group 1 and 2. BDNF expression was significantly reduced in the hippocampus of group 4, compared to group 3. No significant differences in TrkB expression were found; the level of Akt1 expression significantly decreased in the hippocampus in group 4 compared to group 3. No difference was observed in the Morris water maze.
Okihara, H., et al. (2014). *Journal of Neuroscience Research*, *92*(8), 1010–1017.							
Male C57 BL/6J mice [14]	3 wk [powdered diet mixed with water in a 1:4 ratio (group 1); pellet chow (group 2)].	14 wk.	Latency in PAT; number of pyramidal neurons in CA1 and CA3; BDNF and TrkB levels; phospho-44/42 ERK/pan-ERK ratio; body weight.	Cerebral cortex and hippocampus (CA1 and CA3).	Passive avoidance test (PAT); Hematoxilyn staining; Western blotting; body weight measurement.	Not investigated.	Mice in group 1 showed the following characteristics: memory impairment; the level of BDNF was significantly higher and that of TrkB was significantly lower in the hippocampus; the ratio of phospho-p44/42 ERK/pan ERK was significantly lower in the hippocampus and cortex; the numbers of pyramidal neurons in the hippocampus CA1 and CA3 regions of liquid-diet-fed young mice were significantly lower.
Patten, A. R., et al. (2013). *Neuroscience*, *254*, 173–184.							
Male and female Srague-Dawley rats [30]	3 wk [powdered diet mixed with water (group 1, females; group 2, males); pellet chow (group 3, females; group 4, males)].	9 wk (60 d).	Serum corticosterone concentration; number of Ki67-, PCNA-, NeuroD-, DCX- and BrdU-labeled cells; body weight.	Hippocampus and hypothalamus.	ELISA; immunohistochemistry; body weight measurement.	No significant difference in corticosterone concentration among groups.	Liquid diet feeding significantly decreases progenitor cell proliferation in the SGZ of the DG and the PVZ of the hypothalamus; no differences were detected in the density of hippocampal immature neuroblasts using NeuroD and DCX, and cell survival was also unaffected.
Utsugi, C., et al. (2014). *PloS One*, *9*(5), e97309.							
Female C57BL/6 mice [131]	24–28 wk [hard diet for 1 mo (group 1); soft diet for 1 mo (group 2); 1 mo of soft diet then switch to hard diet for 1 mo (group 3); 1 mo of soft diet then switch to hard diet after 3 mo (group 4)].	7–8 mo (group 1 and 2); 8–9 mo (group 3); 11–12 mo (group 4).	Number of BrdU-, c-Fos- and DCX-labeled cells; serum corticosterone concentration; magnitude of preference for 50% butyric acid in Y-maze; body weight.	DG, SVZ, olfactive bulb and principal sensory trigeminal nucleus.	Immunohistochemistry; ELISA; Y-maze apparatus; body weight measurement.	No significant difference was found among groups.	After 1 month, the density of BrdU-ir cells in the SVZ, OB and DG was lower in group 2 than in group 1. Avoidance of butyric acid was reduced in group 2. At 3 months of hard-diet feeding, avoidance of butyric acid was reversed and responses to odors and neurogenesis were recovered in the SVZ (group 4).
Yoshino, F., et al. (2012). *Neuroscience Letters*, *508*(1), 42–46.							
Male Wistar rats [24]	3 wk [regular pellet chow (group 1); powdered diet (group 2)].	12 wk.	DA levels (basal and stimulated); free radicals levels.	Hippocampus.	High performance liquid chromatography; electron-spin resonance (probe: MC-PROXYL); in vitro X-band spectrometry.	Not investigated.	DA release in the hippocampus was decreased in group 2; electron-spin resonance studies directly demonstrated a high level of oxidative stress in the rat brain due to soft-food diet feeding. In addition, it was confirmed that DA directly reacts with reactive oxygen species such as hydroxyl radical and superoxide.

**Table 4:** Molar extraction.

Animal model and [N]	Age at stimulus application and [type of stimulus]	Age at sacrifice	Marker analyzed	Structure analyzed	Type of analysis	Corticosterone evaluation		Results
Aoki, H. et al. (2010) *Neuroscience Letters*, *469*(1), 44–48.								
Male Wistar rats [43].	7 wk (groups 1 and 2) [bilateral extraction of maxillary molars; sham operation (control)].	16 wk (group 1) and 24 wk (group 2).	Brdu-labeled cells; serum corticosterone.	DG.	Immunohystochemical; radioimmunoassy.	In group 2, serum corticosterone was significantly increased.		The molarless condition decreased cell proliferation in the DG.
Iida, S., et al. (2014). *Archives of Oral Biology*, *59*(2), 133–141.								
Male Wistar rats [52].	7 wk [bilateral extraction of maxillary molars (group 1) and subsequent fitting of experimental denture at 11 wk (group 2); sham-operation (control)].	49 wk.	Thyrotropin-releasing hormone (Trh) gene, tenascin XA (Tnxa) gene, neuronatin (Nnat) gene, S100a9 gene; number of errors in the radial arm maze; serum corticosterone.	Hippocampus.	Microarray analysis; eight-arm radial maze; enzyme immunoassay.	No significant difference between groups.		The mean number of errors in the maze was significantly higher for group 1 compared to group 2 and control; Tnxa, Nnat and S100a9 genes may be related to memory function; S100a9 was significantly downregulated in molarless mice (group 1) before maze training when compared to group 2 and control.
Katano, M., et al. (2020). *International Journal of Dental Sciences*, 17 (4), 517–524.								
SAMP8 male mice [14].	4 wk [bilateral extraction of maxillary molars (group 1); sham operation (group 2).	9 mo.	Morphological features of hippocampal neurons (cytoplasm, mitochondria and lipofuscin areas of pyramidal neurons; volume density of mitochondria- and lipofuscin-occupied cytoplasm; number of lipofuscin granules and microtubules per neuron; inner/outer axonal diameter ratio; size of synapses); serum corticosterone; daily food intake and body weight.	Ca1, CA3 and DG.	Cresyl violet staining; TEM microscopy; ELISA; body weight measurement.	In group 1, serum corticosterone was significantly increased.		In group 1, the number and volume of lipofuscin granules was significantly increased, the myelin sheath was significantly thinner and synapse size significantly smaller; there was no significant difference in body weight or food intake.
Kondo, H., et al. (2016). *Archives of Oral Biology*, *61*, 1–7.								
Male SAMP8 mice [80].	32 wk [bilateral extraction of maxillary molars (group 1); sham operation (control)].	35 wk (from all groups, to study newborn cell proliferation, 24 h after Brdu injection) and 38 wk (from all groups, to study survival and differentiation of newborn cells, 21 d after Brdu injection).	Brdu-labeled cells; NeuN- and GFAP-labeled cells; BDNF concentration; escape latency in the Morris water maze;	DG.	Morris water maze test; immunohistochemistry; ELISA.	Not investigated.		Removing the molars of aged SAMP8 mice significantly reduced cell proliferation, newborn cell survival, and cell differentiation in the hippocampal DG, and the hippocampal BDNF protein expression. In addition, the molarless condition impaired the spatial learning and memory in the Morris water maze.
Kubo, K. Y., et al. (2017). *Archives of Oral Biology*, *74*, 21–27.								
Male SAMP8 mice [66].	4 wk [bilateral extraction of maxillary molar teeth (group 1); sham operation (control)].	32 wk.	Serum corticosterone concentration; body weight; escape latency in the Morris water maze; Brdu-, NeuN- and GFAP-labeled cells; Synaptophysin expression.	DG.	Radioimmunoassay; body weight measurement; Morris water maze test; immunohistochemistry; immunoblotting.	In group 1, serum corticosterone was significantly increased.		Aged mice with early tooth loss exhibited hippocampus-dependent learning deficits in the Morris water maze, decreased cell proliferation and cell survival in the dentate gyrus, and suppressed synaptophysin expression in the hippocampus. Newborn cell differentiation in the hippocampal dentate gyrus, however, was not affected by early tooth loss. No significant difference in body weight between groups.
Kurata, C., et al. (2012). Pediatric Dental Journal, 22(2), 110–116								
Male SAMP8 mice [40]	4 wk [bilateral extraction of maxillary molar teeth (group 1); sham operation (control)].	1, 5 and 9 mo.	Escape latency in water maze; serum corticosterone concentration; number of Brdu-labeled neurons in DG.	DG.	Morris water maze test; visible probe test; radioimmunoassay; immunohistochemistry		In group 1, serum corticosterone was significantly increased at 5 and 9 mo.	Plasma corticosterone levels, escape latency in the water maze (but not in the visible probe test) and the number of BrdU-positive cells in the hippocampal DG differed significantly between 5- and 9-mo-old group1 and controls, but not between 1-mo-old group 1 and controls.
Kurozumi, A., et al. (2019). *Journal of Prosthodontic Research*, 63 (3), 283–287.								
Male Wistar rats [30].	7 wk [bilateral extraction of maxillary molars (group 1) and subsequent fitting of experimental denture at 11 wk (group 2); sham-operation (control)].	61 wk.	Number of errors in the radial maze; number of pyramidal neurons; body weight.	CA1 and CA3.	Eight-arm radial maze; Nissl staining; body weight measurement;	Not investigated.		Performance of group 1 and 2 was significantly poorer in the radial maze; the number of pyramidal neurons in group 1 was significantly smaller than group 2 and control in both CA1 and CA3; in group 2, it was significantly smaller than control only in CA1. No significant difference in body weight between groups.
Luo, B., et al. (2019). *Archives of Oral Biology*, 102, 225–230.								
Male Wistar rats [32].	3 mo [bilateral extraction of maxillary molars (group 1); sham operation (group 2)].	6 mo.	Escape latency in water maze; CBF (cerebral blood flow) in the hippocampus; glutammate concentration; Bcl-2 and Caspase-3 mRNA expression; number of pyramidal neurons; body weight.	CA1.	Morris water maze; ASL-MRI (arterial spin labeling magnetic resonance imaging); HPLC (high performance liquid chromatography); rt-PCR; Nissl staining; body weight measurement.	Not investigated.		In group 1, performance in the water maze was significantly poorer; CBF and the number of pyramidal neurons were significantly lower; glutamate concentration, Bcl-2 and Caspase-3 mRNA expression were significantly higher; no significant difference in body weight.
Oue, H., et al. (2013). Behavioural Brain Research, 252, 318–325.								
Female J20 mice [17]	6 mo [bilateral extraction of maxillary molars (group 1); sham operation (control)].	10 mo.	Latency time in STPAT; Abeta40 and Abeta42 concentration; Abeta deposition area; number of CA1 and CA3 pyramidal neurons; body weight; serum corticosterone concentration.	Hippocampus.	Step-through passive avoidance test (STPAT); ELISA; immunohistochemistry; Nissl staining; body weight measurement.	Not investigated.		The molarless condition was associated with a reduction in the number of pyramidal cells, impairing learning and memory abilities, and induced atrophy of pyramidal cells without affecting the Abeta levels; no alteration of serum corticosterone level was found; no significant difference in body weight.
Oue, H., et al. (2016). *Gerodontology*, *33*(3), 308–314.								
Female Tg2576 mice [23]	14 mo [bilateral extraction of maxillary molars (group 1); sham operation (control)].	18 mo.	Latency time in STPAT; Abeta40 and Abeta42 concentration; Abeta deposition area; number of CA1 and CA3 pyramidal neurons; body weight.	Hippocampus.	Step-through passive avoidance test (STPAT); ELISA; immunohistochemistry; Nissl staining; body weight measurement.	Not investigated.		The amount of Ab and the number of pyramidal cells in the hippocampus were not significantly different between the experimental (molarless) and control groups. Similarly, the difference of learning and memory ability could not be distinguished between the group; no significant difference in body weight.
Pang, Q., et al. (2015). *Behavioural Brain Research*, *278*, 411–416.								
Male KM mice [60]	10–11 mo [bilateral extraction of maxillary molars (group 1); sham operation with removal of some bony tissue from the toothless region of the maxillary arch (control 1); bilateral extraction of mandibular molars (group 2) sham operation with removal of some bony tissue from the toothless regione of the mandibular arch (control 2)].	8 wk after stimulus application.	Escape latency, cumulative distance from the platform center, swimming speed in the Morris water maze; NO concentration; iNOS expression;	Hippocampus; cerebral cortex.	Morris water maze test; Griess assay for NO production; immunohistochemistry; Western blot; body weight measurement.	Not investigated.		The molarless condition, both maxillary and mandibular, was associated with impaired spatial learning and memory, as well as increased levels of NO and iNOS in the hippocampus, but not in the cerebral cortex. No significant difference in body weight was found between the groups.
Pang, Q., et al. (2020). *Journal of Dental Sciences*, 15 (1), 84–91.								
Male Wistar rats [48].	3 mo [bilateral ligation of common carotid artery (group 1) and sham operation (group 2); bilateral extraction of maxillary molars (group 3) and sham operation (group 4); no treatment (control).	5 mo.	Escape latency in water maze; NO production; iNOS and eNOS expression; body weight.	Hippocampus.	Morris water maze test; Griess assay; immunohistochemistry; western blot analysis; body weight measurement.	Not investigated.		Groups 1 and 3 had significantly poorer performances in the water maze, higher NO release, more iNOS-positive cells, and fewer eNOS-positive cells than controls, but there was no significant difference between group 1 and 3; no significant difference in body weight.
Sakamoto, S., et al. (2014). *Journal of Oral Rehabilitation*, *41*(10), 715–722.								
Male Wistar rats [30]	7 wk [bilateral extraction of maxillary molars (group 1) and subsequent fitting of experimental denture at 42 wk (group 2); sham-operation (control)].	52 wk.	Number of errors in the maze test; serum corticosterone concentration; neuron density in CA1, CA3 and DG; body weight.	CA1, CA3 and DG.	Radial arm maze test; serum corticosterone (ELISA); Nissl staining; body weight measurement.	No significant difference between groups.		The error incidence in the maze test in group 2 was significantly higher than that of the control group, but significantly lower than that of group 1. Similarly, the neuron density in group 2 was significantly lower than control, but higher than group 1. No significant difference in serum corticosterone levels or body weight between the three groups could be observed.
Takeda, Y., et al. (2016). *BMC Neuroscience*, *17*(1), 1–10.								
Male C57 BL/6J mice [48]	28 wk [bilateral extraction of maxillary molars and solid diet (group 1); bilateral extraction of maxillary molars and powdered diet (group 2); sham operation and solid diet (group 3); sham operation and powdered diet (group 4)].	32 wk (short term experimentation of 4 wk) and 44 wk (long term experimentation of 16 wk).	Latency time in acquisition trials and retention trials in STPAT; BDNF mRNA and TrkB mRNA levels in hippocampus and hypothalamus; number of neurons in CA1 and CA3; body weight.	Hippocampus and hypothalamus.	Step-through passive avoidance test (STPAT); RT-PCR; Nissl staining; body weight measurement.	Not investigated.		At short term experimentation, there were no differences in memory function and BDNF mRNA level between the four groups. However, at long term experimentation, groups 1 and 2 showed memory impairment and decreased level of BDNF mRNA. The powder diet had no effect on memory function and BDNF mRNA level even at 16 wk. The number of neurons was lower in group 1 and 2; at long term experimentation, group 2 showed significantly fewer neurons than group 1 in CA1. After 32 wk, group 2 exhibited significantly lower body weight than group 3.

**Table 5:** Bite-raising.

Animal model and [N]	Age at stimulus application and [type of stimulus]	Age at sacrifice	Marker analyzed	Structure analyzed	Type of analysis	Corticosterone evaluation	Results
Katayama, T., et al. (2012). *Neuroscience Letters*, 520 (1), 77–81.							
Male SAMP8 mice [26]	9 mo [0,1 mm UV-polymerisation resin on maxillary molars (group 1); sham operation (control)].	2 wk after stimulus application.	ACh (acetylcholine) release (hippocampus); number of ChAT (choline-acetyltransferase)-labeled cells (MSN)	Hippocampus and MSP (medial septal nucleus).	In vivo microdialysis; immunohistochemistry; body weight measurement.	Not evaluated (see Discussion)	In group 1, ACh release after KCl perfusion and ChAT-positive cells number were significantly lower. No significant difference in body weight.
Kojo, A., et al. (2010). *The Tohoku Journal of Experimental Medicine*, 221(3), 237–243.							
Male DDy mice [32]	Not declared [0,1 mm UV-polymerisation resin on maxillary molars (group 1); sham operation (control)].	1, 3 and 5 days after stimulus application.	Iba-1 (Ionized calcium-binding adaptor molecule 1, a marker for microglia).	CA1 and DG.	Immunohistochemistry.	Not evaluated (see Discussion)	3 days after stimulus application, in group 1, the area occupied by microglia in the CA1 increased.
Mori, D., et al. (2013). *Neuroscience Letters*, 534, 228–232.							
Male SAMP8 mice [116]	3, 5 and 9 mo [0,1 mm UV-polymerisation resin on maxillary molars (group 1, 3 mo; group 2, 5 mo; group 3, 9 mo); sham operation (age-adjusted control)].	1, 3, 7, 10, and 14 days after stimulus application.	Number of BrdU-labeled cells; escape latency; body weight.	DG.	Immunohistochemistry; Morris water maze test; body weight measurement.	Not evaluated (see Discussion)	In group 3, the number of BrdU-labeled cells was significantly reduced, decreasing abruptly around day 7 and then slowly recovering, without reaching baseline levels; escape latency was significantly increased; no difference in body weight was found after the third post-operative day among experimental groups.
Mori, D., et al. (2016). *Archives of Oral Biology*, 65, 95–101.							
Male SAMP8 mice [114]	9 mo [0,1 mm UV-polymerisation resin on maxillary molars (group 1); sham operation (control)].	1, 7, 14 and 21 days after stimulus application.	Number of BrdU-, NeuN and GFAP-immunoreactive cells; evaulation of apoptosis levels; newborn cell proliferation, survival, differentiation and apoptosis; BDNF mRNA levels; BDNF levels.	DG.	Immunohistochemistry; Terminal deoxyribonucleotidyl transferase dUTP nick-end labeling (TUNEL) assay; in situ hybridization; ELISA.	Not evaluated (see Discussion)	In group 1, neuronal proliferation and survival was significantly reduced; apoptosis levels increased up to day 7 after stimulus application and slowly returned to baseline levels at dau 21; BDNF expression was significantly decreased.
Yamada, K., et al. (2013). *The Tohoku Journal of Experimental Medicine*, 230(1), 49–57.							
Male DDy mice [84]	Not declared [0,1 mm UV-polymerisation resin on maxillary molars (group 1); sham operation (control)].	1, 3 and 5 days after stimulus application.	Dynorphin A.	Hippocampus and amygdala	Immunohistochemistry; ELISA; Morris water maze test.	Not evaluated (see Discussion)	In group 1, dynorphin A levels increased transiently after stimulus application in the amygdala, but not in the hippocampus; escape latency was significantly longer only on day 3 after stimulus application.

**Table 6:** Summary of results.

Experimental condition	Animal model	Body weight and development	Outcome: Histological and biochemical effects	Outcome: Behavioral effects	Outcome: Role of stress
***Soft-diet feeding***	• Mouse (C57BL/6; Albino Swiss; Balb/c; B6C3Fe-a/a; SAMP8; SMR1)	• No significant difference in body weight	• Significant reduction of pyramidal neurons in CA1 and CA3	• Significantly poorer performance, impaired memory and spatial orientation	• No significant difference in corticosterone incretion (2 studies; see Discussion)
		• Significant reduction in craniofacial development	• Significant reduction of neurogenesis in DG		
	• Rat (Wistar; Sprague-Dawley)		• BDNF downregulation		
			• Significant reduction of synaptic density		
			• Astrocyte hyperplasia in CA1		
***Molar extraction***	• Mouse (SAMP8; KM; C57BL/6J; Tg2576; J20)	• No significant difference in body weight	• Significant reduction of pyramidal neurons in CA1 and CA3	• Significantly poorer performance, impaired memory and spatial orientation	• Included studies not in agreement (see Discussion)
		• Significant reduction in body weight when molar extraction and soft-diet feeding were associated	• Significant reduction of neurogenesis in DG		
	• Rat (Wistar)				
			• BDNF downregulation		
			• Significant reduction in synapse size and synaptophysin expression and myelin thickness		
			• Significant reduction in blood flow to the hippocampus		
			• Significant increase in glutamate concentration, apoptosis markers expression and oxidative stress		
***Bite-raising***	• Mouse (SAMP8; DDy)	• No significant difference in body weight	• Significant reduction of neurogenesis in DG	• Significantly poorer performance, impaired memory and spatial orientation	• Included studies did not evaluate (see Discussion)
			• BDNF downregulation		
			• Significant increase of microglia in CA1		
			• Significant reduction in stimulated ACh release and ChAT-positive cells		

Resuming the results of the analytical evaluation of included studies, the literature reviewed agrees that, in the three experimental conditions, disturbed mastication is significantly associated with 1) reduced number of hippocampal pyramidal neurons in CA1 and CA3, 2) downregulation of BDNF (brain derived neurotrophic factor), 3) reduced synaptic activity, 4) reduced neurogenesis in DG, and 5) glial proliferation; these histochemical alterations are associated with reduced performances in behavioural tests that target hippocampus-dependent functions, i.e. spatial orientation and memory. Interestingly, soft-diet feeding and molar extractions were not consistently associated with a stress response.

A few studies indicate that the loss of neurons is partially reversible if correct chewing is restored, by means of either fitting dentures or modifying the consistency of the diet. More in depth, three studies included in their design the extraction of all maxillary molars and the subsequent fitting of experimental dentures. In all instances, the fitting of experimental dentures had positive effects on behavioural performance [24, 28, 34] and histological analysis [28, 34]. In the latter study, the dentures were fitted after 35 weeks of the molarless condition, mimicking the clinical condition of a patient who has been rehabilitated with a removable prosthetic appliance after a long period of time of untreated partial or total edentulism. The experimental dentures group performed less well than control, but significantly better than untreated molarless mice in both the radial arm maze test and histological examination [34].

In addition to the above results, to investigate the effects of a harder-than-usual alimentary bolus on the hippocampus, Akazawa and colleagues [15] hardened regular pellet chow by autoclave cycling, in order to obtain an initial bolus of the same volume and shape, but different compression resistance (which was duly measured and reported). Mice fed a hard diet for 20 weeks presented an increased number of hippocampal neurons, a larger hippocampal volume and performed better in the Morris water maze, when compared to mice fed a regular solid diet. In experimental animals, a hard diet appears to have a protective, or even an enhancing effect on the hippocampus, in terms of both histological findings and behavioral outcomes.

## Discussion

The purpose of this systematic review is to evaluate the evidence coming from animal studies on the relationship between different forms of experimental disruption of the masticatory function and morphofunctional alterations of the hippocampus and behavioural tests. The originality of this paper resides in describing collectively the effects on the hippocampus of three forms of experimentally induced masticatory disturbances: soft-diet feeding, molar extraction and bite-raising. Animal studies shed light on the histological and biochemical alterations together with the behavioural tests associated with experimentally induced masticatory disturbances, thus contributing to an objective understanding of the significance of clinical studies. To our knowledge, a systematic review with these features has not previously been published.

In order to engage in a critical discussion of the topic and to provide reliable conclusions, the included studies have been evaluated with the ARRIVE checklist and the SYRCLE tool for risk of bias in animal studies, as reported in Tables 2 and S1 and S2. Unfortunately, as described in the Results paragraph, it was impossible to adequately assess the risk of selection bias, performance bias and detection bias of included studies with the SYRCLE tool because, with very few exceptions, the necessary information was not reported. Moreover, standard deviations and confidence intervals were not always reported, making it difficult to assess the general precision of the results. On the other hand, the risk of attrition bias, reporting bias or other bias could be adequately assessed with the SYRCLE tool.

As analytically described in the Results, the literature reviewed showed that a disturbed mastication is significantly associated with a reduced number of hippocampal pyramidal neurons in CA1 and CA3, reduced neurogenesis in the DG, reduced synaptic activity, downregulation of BDNF, glial proliferation, and impaired memory and spatial orientation. Indeed, it has recently been shown that hippocampal pyramidal neurons in CA1 reorganize in the course of spatial reward learning [42]. The significance of an observed difference between experimental groups cannot be directly translated to the clinical setting, even though it must be said that a number of clinical studies has already been published in the field. In the context of this review, important aspects of the magnitude of the results are the general agreement among the included studies concerning the histological and biochemical alterations and the proportions of the observed differences, especially concerning the reduction of the number of hippocampal neurons and synaptic activity in conditions of altered mastication, which were generally quite large. Moreover, a correlation was consistently shown between the reduction in the number of hippocampal neurons and reduced performances in behavioural tests targeting hippocampal-dependent cognitive functions (memory and spatial orientation).

Soft-diet feeding protocols have been introduced as a laboratory model of reduced mastication. In this experimental condition, the animal’s occlusion is not altered in any way but the masticatory function, and the muscular activation it entails, is reduced by means of a soft diet, much easier to consume but containing the same nutritional value as the diet of regular, solid consistency that is fed to the control group. This kind of experimental condition is very important because it is free from confounding factors: the occlusion is stable and preserved and it does not significantly alter the animals’ body weight. In a particularly elegant study design, Y. Fukushima-Nakayama and colleagues, using pathogen free mice (further narrowing the possibilities of confounding effects due to inflammation), showed that soft-diet feeding does not alter the growth of the animals’ body but, as would be expected in conditions of impaired masticatory function, it reduces the development of the craniofacial bones and masticatory muscles [2]. Moreover, and unexpectedly, soft-diet feeding was not shown to be a source of stress [20, 21]. Two studies investigated the association between soft-diet feeding and corticosterone incretion: they agreed that soft-diet feeding is not a source of stress, but more research is needed to confirm this important point, which is not yet clear.

Molar extraction protocols suffer from a significant risk of bias due to the necessity of surgically removing the teeth, which may be a source of stress and may cause long lasting infection and inflammation. Furthermore, the extraction of molar teeth introduces local alterations of the occlusion, which may be relevant in conditioning the outcomes. Nevertheless, the results were very close to soft-diet feeding protocols, suggesting that a similar (or the same) biological mechanism is at play. The reports of activation of the HPA axis in the molarless condition were contradictory. The molarless condition was found to be a long-term source of stress by some Authors [23, 25, 27, 36], but not others [24, 34]. Moreover three studies with molar extraction protocols included in their design the fitting of experimental dentures: even though more research is needed on this subject, the results indicate that the rehabilitation of edentulous patients may be beneficial for their cognitive status with a partial recovery of neurons even if the loss of teeth has gone untreated for a long period of time [24, 28, 34].

Bite-raising protocols introduce an extreme form of occlusal disruption that suddenly changes the customary occlusal scheme to two unique prematurities. This form of experimental alteration of the masticatory function is very different from models of reduced mastication with a soft diet: in contrast with the two experimental conditions previously discussed, it has been shown in agreement in studies published before 2010 and therefore not included, but recently reviewed elsewhere [43], to be a source of stress, being significantly associated with increased corticosterone incretion. Humans, as well as animals, are known to release stress by clenching their teeth, i.d. by reaching the occlusal position of maximum intercuspation (MI). When only two premature points of contact are present, as in the bite-raised condition, the dental arches are effectively prevented from reaching MI, which disrupts the physiology of swallowing that occurs with teeth in MI. It is worth underlining that this procedure interferes not only with chewing, but with swallowing and other parafunctional activities of the jaws as well, which differentiates the bite-raised condition from soft-diet feeding and molar extraction models and may be the reason for the activation of the HPA axis in this instance.

Soft-diet feeding models and molar extraction models showed effects in mice of all ages. In SAMP8 mice, a murine model of accelerated senescence, the bite-raised condition was significantly associated with hippocampal alterations especially in aged (9-month-old) animals; however, a positive association was shown in non-aged ddY mice as well [38].

The association between a reduced number of teeth and a greater susceptibility to cognitive impairment in the ageing population is well established [1]; however, clinical studies are at risk of bias, on account of the multiplicity of confounding factors potentially involved. Even though we routinely rely on counting the number of remaining teeth (which is easier to measure clinically and to process epidemiologically), it is quite possible that older people with fewer teeth resemble more closely soft-diet feeding models than they do tooth extraction models. Indeed, people with a suboptimal dentition (generally considered to be less than 20 teeth) tend to eat softer foods, which are easier to chew. Tooth loss in humans recognizes many different causes and, over the length of a human lifetime, it is quite likely that the effects of inflammation and stress associated with tooth extraction or periodontal disease dissipate after a few years.

Soft-diet feeding models may be relevant in young individuals as well. In the industrialized world, softer foods, refined and high in caloric value have become prevalent: the findings of this review strengthen the view that a diet mindful of proper masticatory efficiency may be important to promote the development of the jaws and masticatory muscles as well as to preserve a level of hippocampal neurogenesis adequate to face the challenge of a healthy ageing [44].

The fact that histological and morphological changes in the hippocampus are induced by disruption of masticatory function at any age, after weaning as well as in aged animal models, is of clinical significance. In recent years, the concept of prevention has been associated with that of a repository to be maintained through life: when a biological reserve has been exhausted, prevention is no longer meaningful because the pathological process has already begun, even if it may take years to show itself clinically [45]. The results of this review suggest that the masticatory function may have a role in preserving and continuing stimulating hippocampal trophism, contributing to slow down the gradual cognitive decay associated with old age.

The details of the motor control of mastication are nowadays clear [8], but the influence of mastication on the structure and function of parts of the central nervous system, site of memory and cognition, is probably just beginning to be investigated. Indeed, the most important limitation of this review is that, even though the linkage between impaired mastication (especially soft-diet feeding and, to a lesser extent, molar extraction) and morphofunctional alterations of the hippocampus, together with reduced performances in behavioural tests, is very well established in animals (as well as its association with cognitive status in humans), the biological mechanism responsible for this association is still not clear. A recent study supported the hypothesis that masseter muscle-derived neprilysin is carried along the trigeminus by retrograde axonal transport to reach the hippocampus, following electrical or cholinergic stimulation [46]. More research is necessary to clarify this very important point.

## Conclusions

In conclusion, animal studies significantly substantiate the claim that mastication has a role in maintaining the neuronal population and the synaptic activity of the hippocampus, and, consequently, cognitive performance and memory. The results of this review must be considered in the light of the less than optimal quality of scientific reporting of the included studies, which did not allow a thorough determination of the risk of bias; nevertheless, the consistent reporting of significant histological and functional alterations in conditions of impaired mastication and the agreement with clinical studies suggest with reliability that mastication has a protective role on the CNS and should be preserved free of alterations, as far as possible, from infancy through adulthood and into the aging years. The emerging role of chewing in the preservation of hippocampal trophism, neurogenesis and synaptic activity is fascinating and may contribute to the study of neurodegenerative diseases in new and potentially relevant ways.

## Supporting information

S1 Checklist PRISMA checklist. (DOC)

S1 Table Evaluation of the comprehensiveness of scientific reporting with the ARRIVE guidelines. Evaluation of scientific reporting of included studies by article and by item in the ARRIVE checklist, grouped by type of stimulus.
(XLSX)

S2 Table Evaluation of the risk of bias with the SYRCLE RoB tool. Evaluation of the risk of bias of included studies by article and by item in the SYRCLE RoB tool, grouped by type of stimulus.
(XLSX)

S1 File Summary of search strategy history. Detailed description of the changes made to the search strategy in the course of revision.
(DOCX)
