# Supplementary material for: Altered mastication adversely impacts morpho-functional features of the hippocampus: A systematic review on animal studies in three different experimental conditions involving the masticatory function
Source: PLoS One. 2020 Aug 20;15(8):e0237872. doi: 10.1371/journal.pone.0237872 (PMC7446800; doi:10.1371/journal.pone.0237872)
Supplement: S1 File — Detailed description of the changes made to the search strategy in the course of revision. (DOCX) [file pone.0237872.s004.docx]

**S1 File 1 - Summary of the history of search strategies.**

The search strategy of this systematic review was significantly modified in the late stages of writing, in the course of the peer review process. After the first step of revision, the search strings were modified because of problems with the syntax and the Boolean operators. The revised search strings provided a larger number of results, both because of their improved syntax and the publication of studies that satisfied the inclusion criteria in the months lapsed between the initial search and the revised search. In order to keep the number of included studies manageable, a decision was made at this stage to set a publication date limit at 10 years from the time of writing (July 2020). After the second step of revision, a little residual problem with the syntax was addressed and a suggestion was made to include gray literature in the review process, which had been initially excluded (because of concerns with including material that had not been peer-reviewed). In order to accomplish this, the search was revised and expanded to include three more databases: Scopus, OpenGray and Gray Matters. The search was conducted with similarly constructed search strings; however, finding no results in the last two of the three databases, the absence of relevant results was confirmed with free word searches (not shown). The search of these three databases provided one additional study that satisfied the inclusion criteria.

The date and details of the successive search strategies are reported below in table format.

S1 Table 1 – Search string July 2019

| **Database** | **Search strings** | **Number of results** |
| --- | --- | --- |
| **PubMed** | (((((("Mastication"[Mesh] OR mastication* OR chewing*)))) AND (liquid diet OR soft diet OR hard diet OR powdered diet OR hard bolus OR soft bolus))) AND ("Central Nervous System"[Mesh] OR Central Nervous System* OR Cerebrospinal Axi*) | 33 |
| **PubmMed** | ((((((("Mastication"[Mesh] OR mastication* OR chewing*)) OR ("Tooth Extraction"[Mesh] OR tooth extraction* OR molar extraction*)) OR molarless)) AND ("Central Nervous System"[Mesh] OR Central Nervous System* OR Cerebrospinal Axi*))) AND hippocampus) | 99 |
| **PubMed** | (("Hypothalamus"[Mesh] OR hypothalamus)) OR ((((("Hippocampus"[Mesh] OR hippocampus)))) OR (bite-rais*)) AND ("Mastication"[Mesh] OR mastication* OR chewing*) | 173 |
| **Embase** | ('mastication'/exp OR 'mastication') AND ('central nervous system'/exp OR 'central nervous system') AND (('tooth extraction'/exp OR 'tooth extraction') OR molarless) | 31 |
| **Embase** | (('mastication'/exp OR 'mastication') AND (('hypothalamus'/exp OR 'hypothalamus') OR ('hippocampus'/exp OR 'hippocampus') OR (bite AND rais*))) AND [embase]/lim NOT ([embase]/lim AND [medline]/lim: (5 results): ('mastication'/exp OR 'mastication') AND (bite AND rais*) AND ('central nervous system'/exp OR 'central nervous system') | 51 |

S1 Table 2 – Search strings June 2020

| **Database** | **Search string** | **Number of results** |
| --- | --- | --- |
| **PubMed** | (("Mastication"[Mesh] OR masticat* OR chew*) OR (bite-rais*) OR (("Tooth Extraction"[Mesh] OR tooth extract* OR molar extract*) OR molarless) OR (liquid diet OR soft diet OR hard diet OR powdered diet OR hard bolus OR soft bolus) AND ("Hippocampus"[Mesh] OR hippocampus)) | 167 |
| **Embase** | ('hippocampus'/exp OR hippocampus) AND (('mastication'/exp OR mastic* OR chew*) OR (('tooth extraction'/exp OR 'tooth extract*' OR 'teeth extract*' OR 'molar extract*' OR molarless) OR (('bite rais*' OR bite) AND rais*) OR ('soft diet'/exp OR 'liquid diet'/exp OR 'soft diet' OR 'hard diet' OR 'liquid diet' OR powdered diet))) | 206 |
| **Web of Science** | ((hippocampus) AND ((masticat* OR chew*) OR (bite-rais* OR bite rais* OR tooth extract* OR teeth extract* OR molar extract* OR molarless OR soft diet OR hard diet OR liquid diet OR powdered diet))) | 174 |

S1 Table 3 – Search strings July 2020

| **Database** | **Search string** | **Number of results** |
| --- | --- | --- |
| **PubMed** | (("Mastication"[Mesh] OR masticat* OR chew*) OR (bite-rais*) OR (("Tooth Extraction"[Mesh] OR tooth extract* OR molar extract*) OR molarless) OR (liquid diet OR soft diet OR hard diet OR powdered diet OR hard bolus OR soft bolus) AND ("Hippocampus"[Mesh] OR hippocampus)) | 167 |
| **Embase** | ('hippocampus'/exp OR hippocampus) AND (('mastication'/exp OR mastic* OR chew*) OR ('tooth extraction'/exp OR 'tooth extract*' OR 'teeth extract*' OR 'molar extract*' OR molarless) OR (('bite rais*' OR bite) AND rais*) OR ('soft diet'/exp OR 'liquid diet'/exp OR 'soft diet' OR 'hard diet' OR 'liquid diet' OR powdered diet)) | 206 |
| **Web of Science** | ((hippocampus) AND ((masticat* OR chew*) OR (bite-rais* OR bite rais* OR tooth extract* OR teeth extract* OR molar extract* OR molarless OR soft diet OR hard diet OR liquid diet OR powdered diet))) | 174 |
| **Scopus** | ((hippocampus) AND ((masticat* OR chew*) OR (bite-rais* OR bite rais* OR tooth extract* OR teeth extract* OR molar extract* OR molarless OR soft diet OR hard diet OR liquid diet OR powdered diet))) | 98 |
| **OpenGray** | ((hippocampus) AND ((masticat* OR chew*) OR (bite-rais* OR bite rais* OR tooth extract* OR teeth extract* OR molar extract* OR molarless OR soft diet OR hard diet OR liquid diet OR powdered diet))) | 0 |
| **GrayMatters** | ((hippocampus) AND ((masticat* OR chew*) OR (bite-rais* OR bite rais* OR tooth extract* OR teeth extract* OR molar extract* OR molarless OR soft diet OR hard diet OR liquid diet OR powdered diet))) | 0 |
